# Nucleolar TRF2 attenuated nucleolus stress-induced HCC cell-cycle arrest by altering rRNA synthesis

**DOI:** 10.1038/s41419-018-0572-3

**Published:** 2018-05-03

**Authors:** Fuwen Yuan, Chenzhong Xu, Guodong Li, Tanjun Tong

**Affiliations:** Research Center on Aging, Department of Medical Biochemistry and Molecular Biology, School of Basic Medical Sciences, Peking University, Beijing, China

## Abstract

The nucleolus is an important organelle that is responsible for the biogenesis of ribosome RNA (rRNA) and ribosomal subunits assembly. It is also deemed to be the center of metabolic control, considering the critical role of ribosomes in protein translation. Perturbations of rRNA synthesis are closely related to cell proliferation and tumor progression. Telomeric repeat-binding factor 2 (TRF2) is a member of shelterin complex that is responsible for telomere DNA protection. Interestingly, it was recently reported to localize in the nucleolus of human cells in a cell-cycle-dependent manner, while the underlying mechanism and its role on the nucleolus remained unclear. In this study, we found that nucleolar and coiled-body phosphoprotein 1 (NOLC1), a nucleolar protein that is responsible for the nucleolus construction and rRNA synthesis, interacted with TRF2 and mediated the shuttle of TRF2 between the nucleolus and nucleus. Abating the expression of NOLC1 decreased the nucleolar-resident TRF2. Besides, the nucleolar TRF2 could bind rDNA and promoted rRNA transcription. Furthermore, in hepatocellular carcinoma (HCC) cell lines HepG2 and SMMC7721, TRF2 overexpression participated in the nucleolus stress-induced rRNA inhibition and cell-cycle arrest.

## Introduction

The function of gene is regulated in many ways, including protein production, modification, distribution, and degradation^1,2^, among which the regulation of protein distribution between different subcellular organelles is one important way^3–5^. The sub-organelles regulation role of the nucleolus, a eukaryotic subnuclear organelle, which is responsible for ribosomal RNA transcription, processing, modification, and ribosomes assembly, was recently reported frequently^6–8^. Accumulating evidences have linked this organelle to many other aspects except for ribosome RNA (rRNA) metabolism, leading to the concept of plurifunctional nucleolus^9–14^. More and more evidence demonstrated that telomeric components such as telomeric repeat-binding factor 1 (TRF1) and telomerase are also localized in the nucleolus of mammalian and yeast cells in these years^12,15,16^. Recently, we and some other groups have found that telomeric repeat-binding factor 2 (TRF2) was localized in the nucleolus in HEK293T, MCF7, and some other hepatocellular carcinoma (HCC) cells, while the role of nucleolar TRF2 remains unclear^17–19^.

Nucleolar and coiled-body phosphoprotein 1 (NOLC1) is a nucleolar protein localized in nucleolar-dense fiber components (DFCs), which also functions as a chaperone for shuttling between the cytoplasm and nucleolus^20,21^. Ubiquitylation NOLC1 could drive the formation of a treacle ribosome biogenesis factor 1 (TCOF1)-NOLC1 platform that remodeled the translational program of differentiating cells in favor of neural crest specification^22^, and it could also act as a transcriptional regulator and activated the alpha-1-acid glycoprotein (agp) in mammalian livers^23^. In addition, hNOLC1 has been demonstrated to function as a binding target of doxorubicin, which is a widely used anticancer drug^24^. As a nucleolar protein, NOLC1 participated in the regulation of rRNA transcription by interacting with the largest subunit of RNA Pol I (RPA194)^25^. Enhanced NOLC1 regulated the distribution of some nucleolus proteins that is responsible for rRNA synthesis and thus perturbed the rRNA processing^12^.

TRF2 coats the full-length of all human telomeres and binds directly to the duplex TTAGGG repeats^26^. The human telomeres protection crucially depends on this factor and we can also assume that the requirement for duplex TTAGGG repeats at chromosome ends reflects the need for TRF2 binding. The early research on TRF2 was primarily focused on its roles in telomere protection and DNA damage repair. Although recent studies have found that TRF2 could also localize in the nucleolus in some human cells in a cell-cycle-dependent manner, the underlying mechanism remained unclear^17,18^. Here, we found that NOLC1 regulated the nucleolus accumulation of TRF2 and the nucleolus accumulated TRF2-promoted rRNA transcription.

## Results

### TRF2 interacted with NOLC1 and accumulated in the nucleolus

**Fig. 1:**
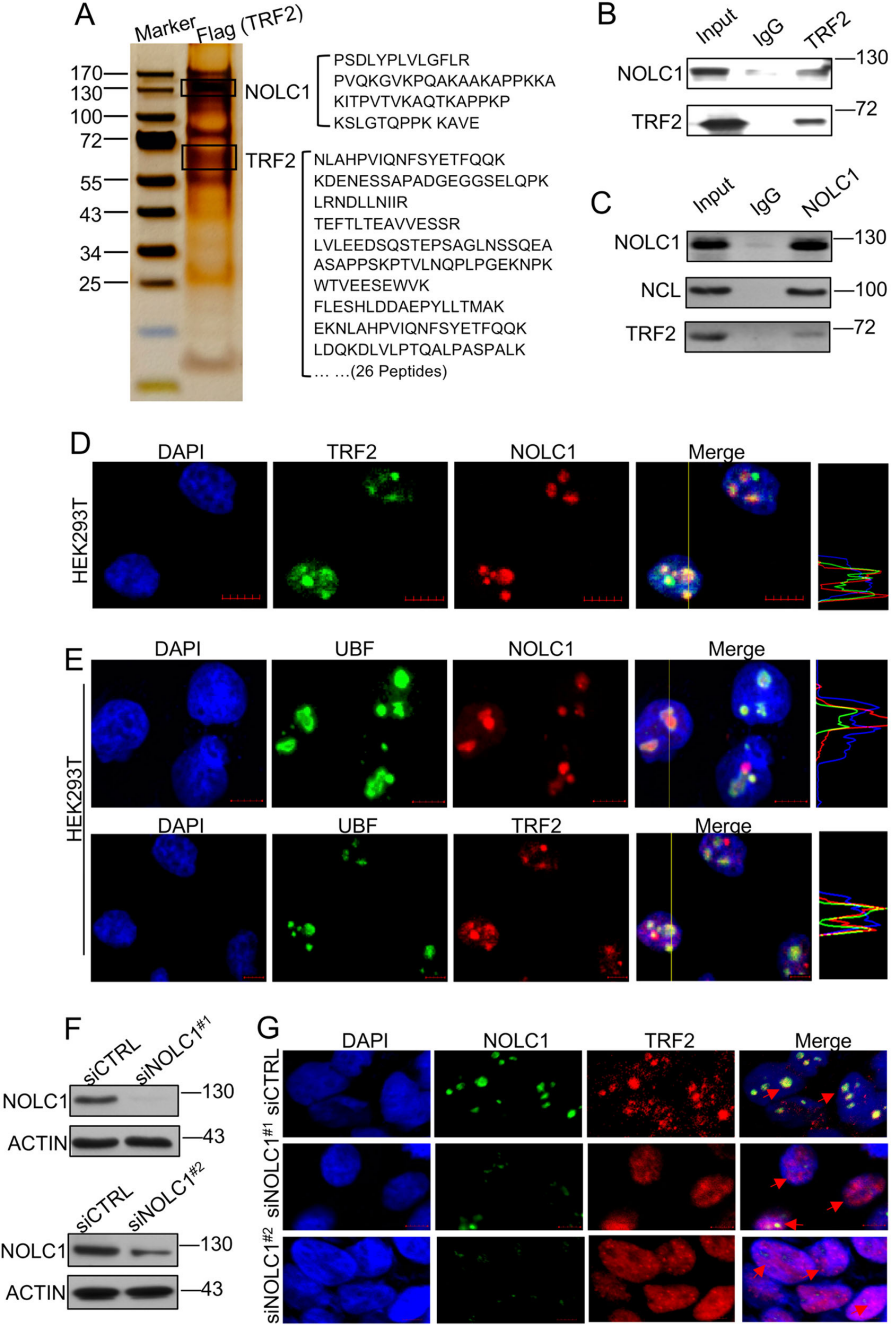
TRF2 interacted with NOLC1 in 293T cells and colocalized in the nucleolus. **a** *In vivo* Flag-tag pull-down analysis. Whole-cell extracts of 293T cells transfected with Flag-TRF2 were obtained with anti-Flag M2 beads followed by mass-spectrometric peptide sequencing. Both TRF2 and NOLC1 were identified. **b**, **c** Reciprocal examination of the physical interaction between NOLC1 and TRF2. Immunoprecipitates obtained using an anti-TRF2 or anti-NOLC1 antibody were subjected to western blot analysis. NCL was characterized as a positive control that interacts with NOLC1. **d** Immunofluorescence analysis revealed the nucleolar colocalization of TRF2 (green) and NOLC1 (red) in human HEK293T. **e** Immunofluorescence analysis of the localization of NOLC1 (red) and UBF (green) in HEK293T cells (upper line) and the colocalization of TRF2 (red) with UBF (green). **f** HEK293T cells were transfected with NOLC1 targeting siRNA or control siRNA (siCTRL), and western blot analyzed the relative expression of NOLC1. **g** The distribution of TRF2 (red) and NOLC1 (green) was observed with immunofluorescence analysis after 72 h of NOLC1 siRNA transfection. Scale bar, 5 μm

In our previous study with mass spectrometry (MS) analysis, we have found that TRF2 was identified in the NOLC1 co-precipitation^19^. To further explore the interaction of TRF2 and NOLC1, we constructed TRF2 expression plasmid with Flag tag and was transfected into HEK293T cells for MS analysis from where NOLC1 was identified (Fig. 1a). Furthermore, endogenous NOLC1 was found in the immunoprecipitation assay with anti-TRF2 antibody (Fig. 1b). Conversely, endogenous TRF2 was detected in the immunoprecipitate obtained from HEK293T cell lysate using an anti-NOLC1 antibody, and nucleolin (NCL) was detected as a positive control of NOLC1-interacting protein^27^ (Fig. 1c).

We further identified the colocalization of endogenous TRF2 and NOLC1 in HEK293T (Fig. 1d and Figure S1A) and hepatoma carcinoma cell SMMC7721 (Figure S1B, first line), but not in cervical cancer HeLa cells (Figure S1B, second line), which was consistent with our previous findings, although we have not found a reasonable explanation so far for the cell-line differences. Additionally, we detected the distribution of NOLC1 and TRF2 with upstream-binding factor (UBF), a nucleolus marker, which further convinced that TRF2 was mostly localized in the nucleolus (Fig. 1e and Figure S1C). We also found that after NOLC1 knockdown, TRF2 was released from the nucleolus into the nucleoplasm (Figs. 1f, g). In order to further investigate whether exogenous TRF2 was also modulated by NOLC1, we next visualized the subcellular distribution of GFP–TRF2 with/without NOLC1 knockdown, from where we can find that most of overexpressed GFP–TRF2 was colocalized with NOLC1 in the nucleolus. TRF2 was diffused into the nucleoplasm after NOLC1 knockdown (Figure S2). Taken together, our results showed that TRF2 was accumulated in the nucleolus by interacting with NOLC1.

### TRF2 knockdown inhibited rRNA expression and was rescued by NOLC1

**Fig. 2:**
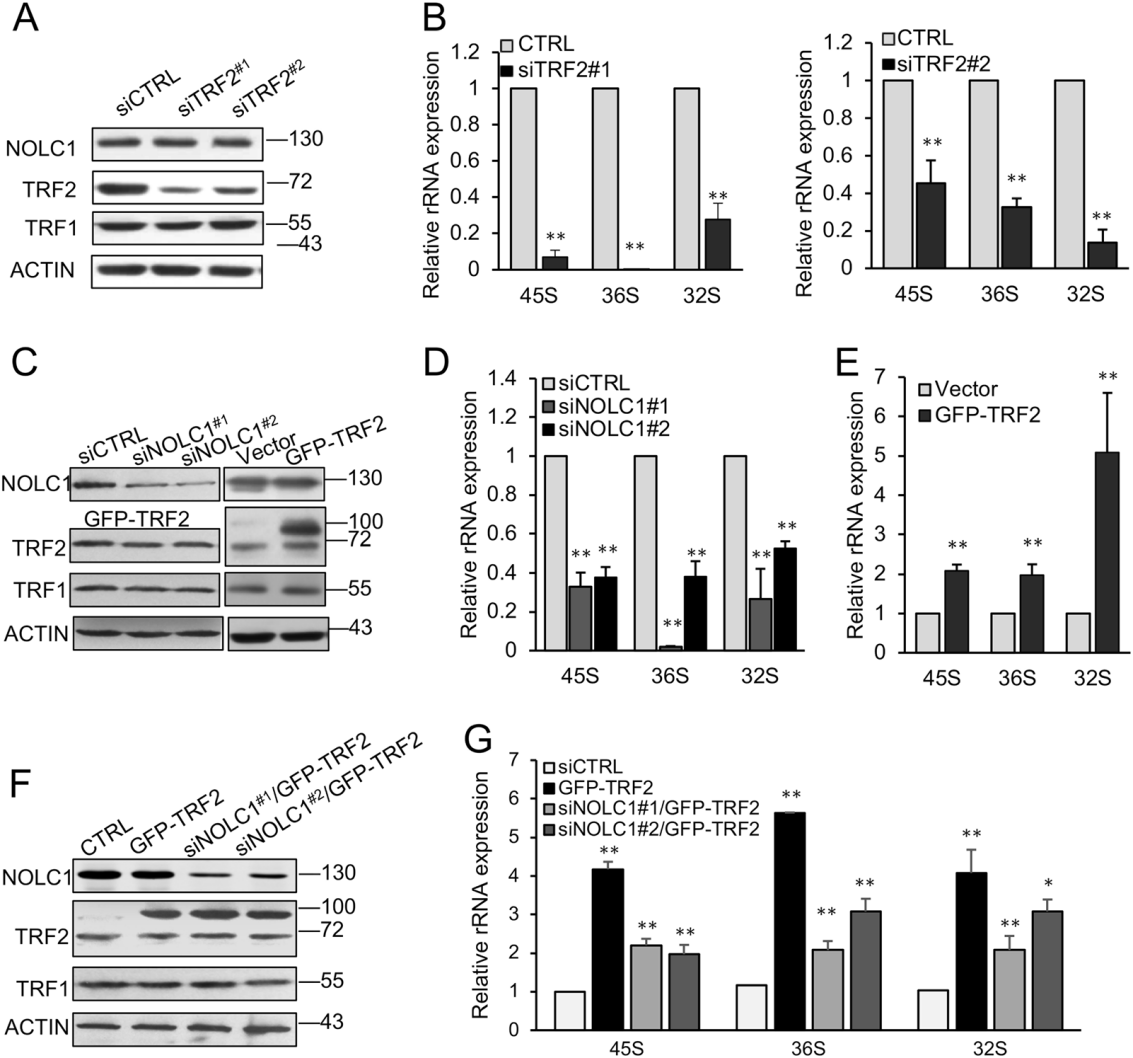
NOLC1 mediated the nucleolar accumulation of TRF2 and affected rRNA expression. **a** Total cell lysates were subjected to western blot for analysis of the knockdown efficiency of TRF2, and TRF1 was detected for ruling out the possible affection of TRF2 siRNA on TRF1. **b** The relative expression levels of 45S, 36S, and 32S rRNA were quantified with real-time PCR after TRF2 knockdown. **c** Western blot analyzed the knockdown efficiency of NOLC1 siRNA and overexpression of GFP–TRF2. **d** Real-time PCR measured the relative expression levels of 45S, 36S, and 32S rRNA after NOLC1 knockdown. **e** The relative expression of 45S, 36S, and 32S rRNA after TRF2 overexpression. **f** Total cell lysates were subjected to western blot analysis of the knockdown efficiency of NOLC1 and overexpression of GFP–TRF2 with indicated antibodies. **g** Quantification of the relative expression levels of 45S, 36S, and 32S rRNA transiently expressing GFP–TRF2 or accompanied with NOLC1 targeting siRNA. Data are presented as the mean ± SD of three independently performed experiments. *p < 0.05, **p < 0.01

Considering the critical role of the nucleolus on rRNA synthesis, we next wondered if TRF2 has any impact on rRNA expression. With real-time PCR analysis, we found that the expression of precursor rRNA was significantly decreased after TRF2 knockdown (Figs. 2a, b), which was the same with NOLC1 knockdown (Figs. 2c, d). Conversely, the rRNA level was increased after TRF2 overexpression (Figs. 2c, e).

In order to further demonstrate if the rRNA expression regulated by TRF2 was dependent on NOLC1, we ablated the expression of NOLC1 after TRF2 overexpression, from where we found that knockdown of NOLC1 could repress the increased rRNA level induced by TRF2 overexpression (Figs. 2f, g), which also indicated that TRF2 regulated the rRNA expression dependent on TRF2 nucleolus localization.

### TRF2 bound rDNA repeats and was regulated by NOLC1

**Fig. 3:**
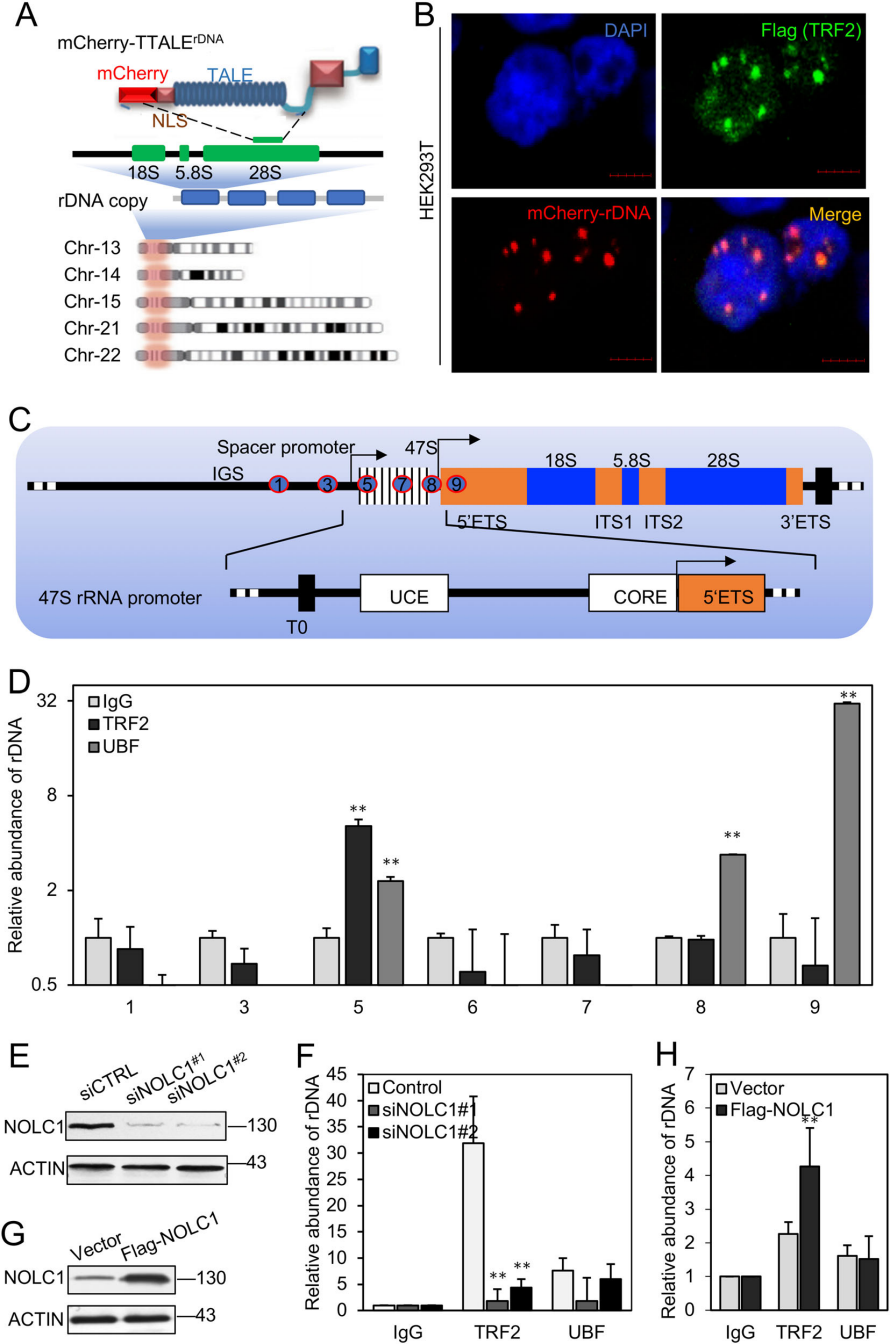
TRF2 bound rDNA in HEK293T cells and was regulated by NOLC1. **a** Schematic illustration of TALEs labeling rDNA, as well as the distribution and structural features of NOR-rDNAs in the human genome. **b** Flag-TRF2, as well as mCherry-rDNA TALE system were transfected in HEK293T cells. Immunofluorescence analysis was performed after 48 h using the Flag antibody to investigate the distribution of Flag-TRF2 (green) and rDNA (red). Nuclei were visualized with DAPI (blue) staining. Scale bar, 5 μm. **c** The rDNA sequence diagram and the exact location of the primers were used for ChIP. **d** The relative abundance of rDNA immunoprecipitated by TRF2 antibody in HEK293T cells. **e**, **g** Total cell lysates were subjected to analyze the knockdown efficiency of NOLC1 and overexpression of NOLC1. **f** The relative abundance of rDNA immunoprecipitated by TRF2 antibody in 293T cells after NOLC1 knockdown. **h** The relative abundance of rDNA immunoprecipitated by TRF2 antibody in 293T cells after NOLC1 overexpression. Data are presented as the mean ± SD of three independently performed experiments. **p < 0.01

It has been reported that NOLC1 could recruit some proteins to the rDNA promoter and regulate the rRNA transcription^25^. We wonder if TRF2 was localized with rDNA. An engineered thioredoxin-fused TALE (TTALE) system^28^ that specifically marked rDNA (Fig. 3a and Figure S3) was transfected with Flag-TRF2, and fluorescence microscopy analysis revealed that Flag-TRF2 colocalized with rDNA in HEK293T cells (Fig. 3b).

In mammals, a single rDNA unit contains a transcribed region followed by intergenic spacer (IGS), which transcripts into precursor 47S rRNA, which is processed to give 18S, 5.8S, and 28S rRNAs^6–8^. In order to further demonstrate the rDNA-binding ability of TRF2, we carried out chromatin immunoprecipitation (ChIP) with a series of primers targeting different regions of rDNA N-terminal (Fig. 3c). The antibody of UBF was used as well, which has been reported previously, and is enriched at the promoter and the transcribed regions of rDNA^29^. Our results indicate that TRF2 binds to the transcription start position of rDNA (Fig. 3d).

In order to determine whether the rDNA-binding ability of TRF2 was affected by NOLC1, we knockdown NOLC1 in HEK293T cells and carried out ChIP. The primer no. 5 was used for measurement of the relative abundance of TRF2 on rDNA, which showed that the relative abundance of rDNA with TRF2 decreased after NOLC1 knockdown (Figs. 3e, f), whereas NOLC1 overexpression increased the binding of TRF2 and rDNA (Figs. 3g, h).

### Truncated TRF2 repressed the expression of rRNA

**Fig. 4:**
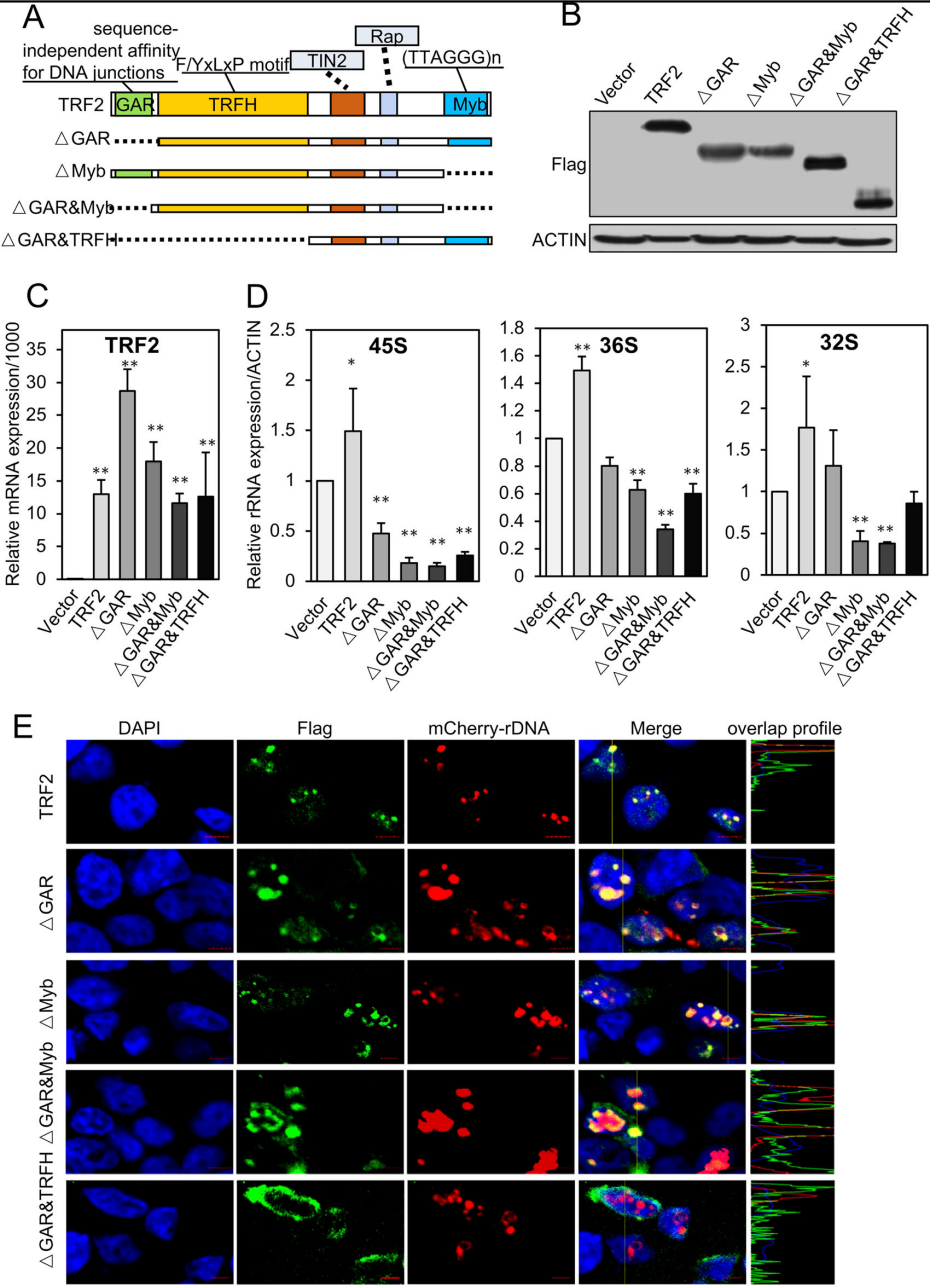
The domain-deleted TRF2-disturbed rRNA expression and rDNA advanced structure. **a** Schematic diagrams of different domain-deleted TRF2 constructed into pcDNA3.1 plasmids. **b** Western blot was performed to analyze the truncated TRF2 proteins expression. **c** The relative mRNA expression of the domain-deleted TRF2 after transfection in 293T cells. **d** The relative expression of precursor rRNAs after transfection of the domain-deleted TRF2 in HEK293T cells for 48 h. **e** Immunofluorescence visualized the protein expression of domain-deleted TRF2 and the distribution of the TRF2 with rDNA. HEK293T cells were transfected with mCherry-rDNA TALE system and Flag-TRF2 or the other Flag-tagged mutant TRF2 for 48 h. The TRF2 (red) and rDNA foci (red) in the nuclei of HEK293T were visualized with the indicated antibodies. Nuclei were visualized by DAPI staining. Scale bar, 5 μm. Data are presented as the mean ± SD of three independently performed experiments. *p < 0.05; **p < 0.01

TRF2 consists of the TRF homology (TRFH) domain and a C-terminal SANT/Myb DNA-binding domain, which are connected through a flexible hinge domain, preceding the TRFH domain, and contains a Gly/Arg-rich domain (GAR domain). The SANT/Myb domains of TRF2 are nearly identical and confer specificity for T in ds DNA. TRF2 binds DNA as homodimers or oligomers formed through homotypic interactions in the TRFH domain^30^. To further explore the critical domain that is responsible for rRNA synthesis, we next expressed the full-length and other truncated TRF2 constructs (Fig. 4a) in HEK293T cells (Figs. 4b, c). The expression of precursor rRNA, including 45S, 36S, and 32S rRNA was quantified (Fig. 4d). These results indicated that either the GAR or Myb domain could inhibit the expression of rRNA, which inferred that both the N-terminus and C-terminus are critical for the role of TRF2 on rRNA synthesis.

Previous study has found that TRF2 is critical for telomere DNA high-ordered structure maintainance, and both the GAR domain and Myb domain are indispensable for its ability of acting as the architectural factor^30^. Here, we found that after we transfected truncated TRF2 in cells, the distribution of mCherry-rDNA became larger and dispersed, which inferred that overexpression of TRF2 without either GAR domain or Myb domain might induce the break and lose the rDNA structure (Fig. 4e and Figure S4), as the other researchers found in telomere. From these results, we suppose that the nucleolus TRF2 might act as a maintenance factor of rDNA structure expression, and the truncated TRF2 disturbed the interaction of TRF2 and rDNA, thus destroying the rDNA structure and inhibiting the expression of rRNA.

### Nucleolus stress induced TRF2 released to the nucleoplasm and repressed rRNA transcription

**Fig. 5:**
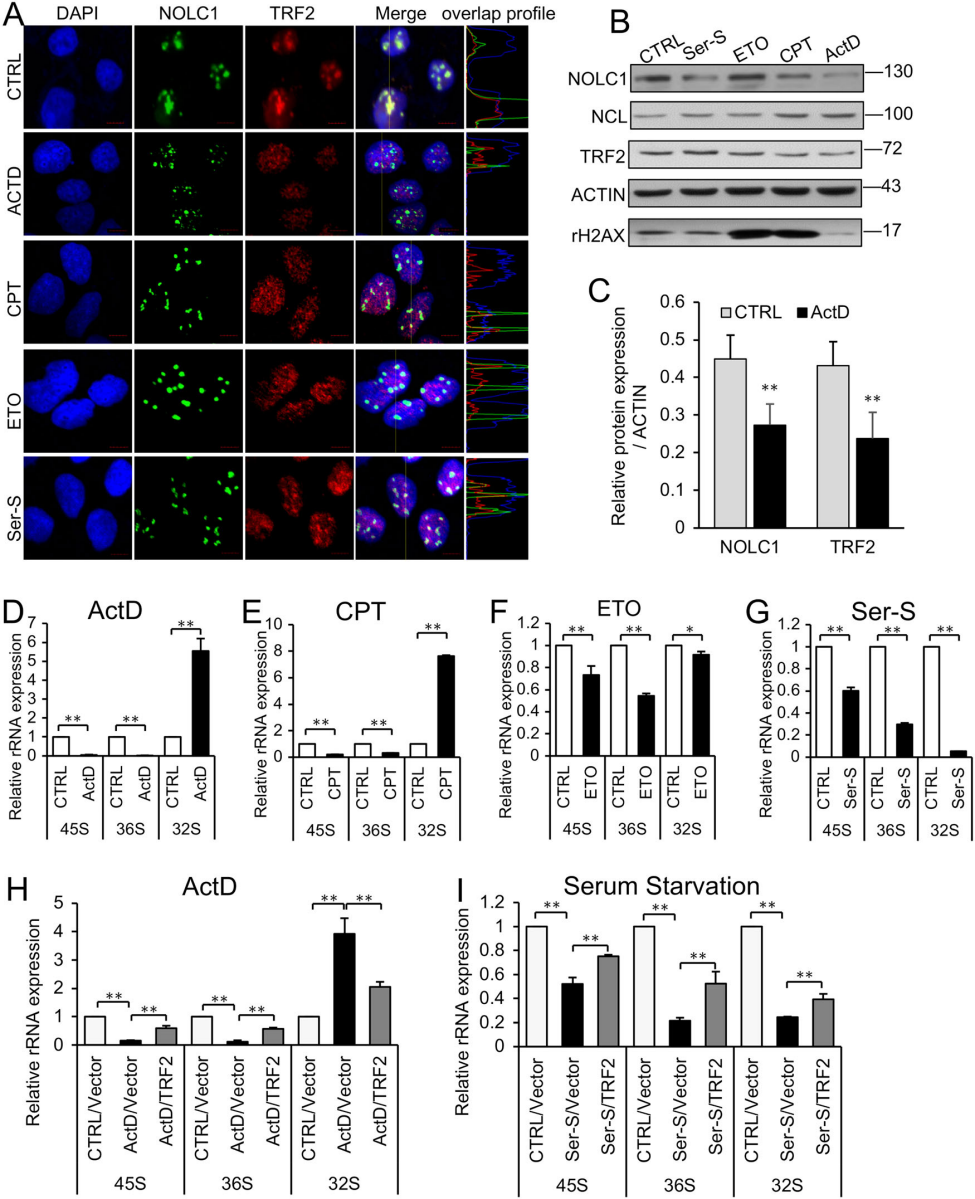
Nucleolar stress dysregulated the colocalization of TRF2 and NOLC1 and inhibited the expression of rRNA. **a** The localization of TRF2 and NOLC1 in HEK293T cells after treatment with actinomycin D (ActD, 5 nM, 6 h), camptothecin (CPT, 20 nM, 6 h), etoposide (ETO, 40 nM, 6 h), and serum starvation (Ser-S, 0.1% FBS, 24 h). **b** Western blotting assay showed the protein level after the HEK293T cells were treated with nucleolar stresses. **c** Relative expression levels of NOLC1 and TRF2 proteins. **d–g** The expression of pre-rRNA in 293T cells after the cells were treated with ActD, CPT, ETO, and serum starvation separately. **h**, **i** The expression of pre-rRNA in HEK293T cells after cells were treated with ActD or serum starvation (Ser-S), whereas overexpression of TRF2 rescued the nucleolar stresses induced by rRNA synthesis repression. Data are presented as the mean ± SD of three independently performed experiments. *p < 0.05, **p < 0.01

Cells respond to numerous stresses, including actinomycin D (ActD), serum starvation, and some DNA damage treatments by disturbing rRNA synthesis. We wondered if TRF2 takes part in the nucleolus stress-induced rRNA transcription inhibition. Indeed, as cells were treated with ActD or in some other nucleolus stress conditions, the distribution changed the amount of TRF2 that colocalized with decreased NOLC1 and was mainly distributed in the nucleoplasm but not nucleolus (Fig. 5a and Figure S5A); besides, the number of NOLC1 and TRF2 foci increased significantly (Figure S5B and C). The expression of TRF2 or NOLC1 has no significant correlated change (Fig. 5b) except the ActD treatment (Figs. 5b, c and Figure S6).

We next detected the rRNA level after HEK293T cells were treated with ActD (5 nM, 6 h), camptothecin (CPT, 20 nM, 6 h), etoposide (ETO, 40 nM, 6 h), and serum starvation (0.1% fetal bovine serum (FBS), 24 h; Figs. 5d–g), and we found that the precursor rRNAs 45S and 36S were decreased after these treatments. Although after ActD and CPT treatment, the 32S rRNA was increased. The rRNA synthesis is a series of processes, which includes pre-rRNA transcription, processing, and mature rRNA assembly with ribosomal proteins (Figure S7). We suppose that it is because ActD and CPT treatment disturbed both the transcription and processing of rRNA, so the 45S and 36S rRNA decreased, while the processing of middle precursor 32S rRNA was dilated and accumulated.

### Overexpression of TRF2 rescued the nucleolus stress-induced rRNA inhibition and promoted HCC cell-cycle arrest

To further figure out if TRF2 released from the nucleolus was correlated with stress-induced rRNA expression inhibition, we overexpressed TRF2 in HEK293T cells, and treated with ActD, or serum starvation after transfection for 48 h. Real-time PCR was carried out and the results indicated that TRF2 overexpression could partly rescue nucleolus stress-induced rRNA expression depression (Figs. 5h, i).

**Fig. 6:**
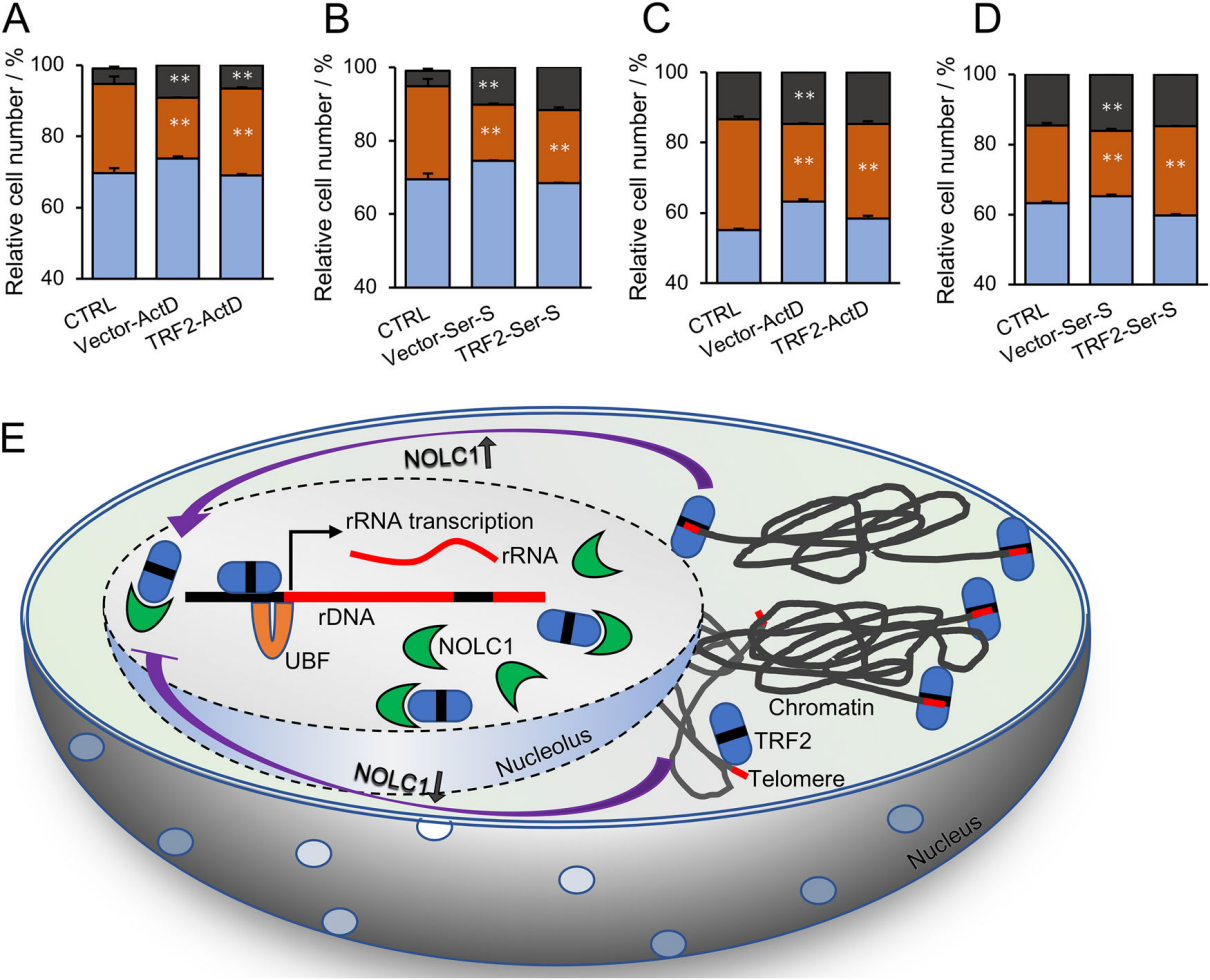
TRF2 overexpression rescued ActD and serum starvation-induced HCC cell-cycle arrest. **a** MMC7721 cells were transfected with TRF2 for 48 h, the cells were then treated with ActD for 10 h, and subjected to flow cytometry cell-cycle analysis. S- and G2-phase arrest was induced following ActD treatment, and the overexpression of TRF2 could partly rescue the cell-cycle arrest. The histogram is the statistics cell cycle. **b** SMMC7721 cells were transfected with TRF2 for 48 h and were treated with DMEM (0.1% FBS) for 24 h and flow cytometry cell-cycle analysis was performed. **c**, **d** HepG2 cells were used for cell-cycle analysis and was performed as described in **a** and **b**. The histogram is the statistics of the cell cycle. All values are presented as mean ± SD of at least three independent experiments; **p < 0.01. **e** A proposed working model to explain how NOLC1 functions in the nucleolar accumulation of TRF2 and the effects of TRF2 on rRNA synthesis and cell-cycle regulation. Under normal conditions, the levels of TRF2 in the nucleolus and at telomeres are strictly balanced. When NOLC1 expression increases, TRF2 accumulates in the nucleolus and promotes rRNA synthesis, which promotes cell-cycle progression

Ribosome biogenesis drives cell growth and proliferation. Perturbation of rRNA transcription or processing induced by nucleolus stress can arrest cells at G1–G1/S or G2/M phase^29^. Here, we detected that the cell-cycle progression of HCC cell lines HepG2 and SMMC7721 were treated with ActD for 12 h or treated for 24 h with serum starvation, from where we found that the cells were significantly arrested in S phase. Overexpression of TRF2 could rescue the ActD or serum starvation-induced cell-cycle arrest (Figs. 6a–d and Figure S8)^31^.

## Discussion

In higher eukaryotes, the interphase nucleoli sustain a tripartite structure: the fibrillar center (FC), DFC, and the granular center (GC). FCs contain inactive rDNA that is mainly for rDNA transcription, which also contains many transcription factors such as RNA polymerase I, topoisomerase I, and UBF^32–34^. The DFC consists of pre-rRNA and early processing factors and surrounds the FCs^35^, whereas late processing factors and ribosomal proteins mainly reside at the GC, where ribosomal assembly takes place^36^.

Recent studies have found that human TRF2 localizes in the nucleolus except its telomere binding; however, if TRF2 participates in rRNA synthesis remains unclear. Here, we found that TRF2 interacted with the nucleolar protein NOLC1 and colocalized with it in the nucleoli of human 293T and HepG2 cells. The nucleolus accumulated TRF2-bound rDNA and promoted rRNA transcription. Besides overexpression, either the GAR domain or Myb domain-deleted TRF2 seemed to induce the loss of the rDNA structure, and the expression of pre-rRNA was decreased, especially loss of Myb domain.

It has been reported that perturbation of nucleolar homeostasis is known to trigger a prompt inhibition of rRNA synthesis and arrest of cell-cycle progression^37–40^. Here, we found that the cell-cycle progression was arrested after the cells were treated with serum starvation or ActD in HCC cell lines HepG2 and SMMC7721. Co-expression of TRF2 rescued nucleolus stress-induced cell-cycle arrest.

The nucleolar accumulation of some proteins such as MDM2 proto-oncogene is regulated by more than one nucleolus protein. Hence, some other nucleolar proteins may also participate in the nucleolar retention of TRF2. In fact, in our MS analysis, we have found that some other nucleolar proteins such as NCL also interacts with TRF2 although further investigations are required. Furthermore, it would be interesting to investigate whether the other telomere-related proteins are also regulated by nucleolus proteins and if any other telomere-related proteins also take part in the regulation of rRNA transcription, in addition to TRF2 and telomerase. In summary, our study demonstrated that the nucleolus protein NOLC1 regulates nucleolar accumulation of telomere-binding protein TRF2. The nucleolar TRF2 binds rDNA and participated in rRNA transcription, and thus takes part in nucleolus stress-induced rRNA synthesis inhibition and HCC cell-cycle arrest (Fig. 6e). Our research suggests another potential role of TRF2 on rRNA transcription, except telomere protection and DNA damage repair functions.

## Materials and methods

### Cell culture, antibodies, and plasmids

The human embryonic kidney cell line HEK293T, the human hepatoma cell line HepG2 and 7721 cells, and HeLa cells were cultured in Dulbecco’s modified Eagle’s medium supplemented with 10% fetal bovine serum, in 5% CO_2_ at 37 °C. The antibodies used in this study were as follows: anti-TRF2 (ab13579, Abcam, Massachusetts, USA), anti-NOLC1 (sc-374033, Santa Cruz Biotechnology, California, USA and ab184550, Abcam, Massachusetts, USA), anti-Flag (F1804, Sigma, California, USA), UBF (sc-9131, Santa Cruz Biotechnology, California, USA), and anti-actin (SC-130300, Santa Cruz Biotechnology, California, USA). The GFP–TRF2 expression plasmid was purchased from Addgene (ID no. 19798). The Flag-NOLC1 expression vector was constructed by inserting the full-length NOLC1 complementary DNA (cDNA) into the pCMV-Flag vector. The Flag-TRF2 and mutant TRF2 expression vectors were constructed by inserting the corresponding TRF2 cDNA into the pcDNA3.1 vectors. The primers were listed in Table S1.

### Flag-tag affinity purification of TRF2 and associated proteins

MS was performed as described previously^27^. Briefly, HEK293T cells were seeded to 4 × 15-cm culture dishes, and when cells were grown to 60% confluence, cells were transfected with Flag-TRF2 and cultured for another 48 h. Cells were then harvested and proteins were extracted by immunoprecipitation lysate buffer (50 mM Tris-HCl, pH 7.4, 150 mM NaCl, 1 mM EDTA, 1 mM DTT, 0.25 mM phenylmethanesulfunyl fluoride (PMSF), 0.3% NP-40, and a cocktail). The bait proteins and their associated proteins were affinity purified using the Flag M2 beads and eluted using the Flag peptide. The eluates were resolved by sodium dodecyl sulfate (SDS)–polyacrylamide gel electrophoresis (PAGE) and visualized by silver staining. The protein bands were retrieved and analyzed by MS.

### Quantitative PCR (real-time PCR)

The total tissue RNA was extracted according to the manufacturer’s protocols, and the first-strand cDNA was synthesized with cDNA synthesis kit (Transgen Biotec Co. Ltd., Beijing, China). Quantitative PCR was performed in triplicate by using the SYBR Green PCR master mix (ABI, Massachusetts, USA) on an ABI 7500 Real Time PCR System. Actin was used as an endogenous control, and fold changes were calculated by means of relative quantification (2^−∆∆ct^). The primers used for quantitative PCR were listed in Table S2.

### Immunofluorescence

Immunofluorescence was performed as described previously^27^. Cells were seeded and grown on coverslips for indicated times. After being washed with phosphate-buffered saline (PBS), cells were fixed for 10 min in 4% paraformaldehyde, incubated for 5 min in 0.5% Triton X-100, then blocked in blocking solution (5% bovine serum albumin prepared in PBST), and incubated overnight at 4°C with primary antibodies prepared in blocking solution. After being washed twice with PBS for 5 min, the cells were then incubated with a secondary antibody conjugated with 647-Alexa (red) or 488-Alexa (green) (Molecular Probes, Oregon, USA). 4', 6-diamidino-2-phenylindole (DAPI) was used as a nuclear stain. Olympus FV1500 confocal microscope was used for microscopic analyses.

### Immunoprecipitation and western blotting

Cells were collected in immunoprecipitation (IP) lysate buffer, 5% of the total protein was kept as input, and the others were incubated with indicated antibodies overnight at 4 °C and protein-G Sepharose beads (Millipore, Darmstadt, Germany) for another 2 h. Then, the immunoprecipitates were washed with IP lysate buffer four times and resuspended in 30 μl of 2 × SDS loading buffer and boiled for 10 min at 100 °C. After a short centrifuge, the supernatant was then used for western blot analysis. For western blot, proteins were extracted and separated by SDS–PAGE, and then they were transferred to a nitrocellulose filter membrane. After being blocked with 5% nonfat milk for 1 h at room temperature, the membrane was incubated with primary antibodies overnight at 4 °C. After being washed twice with PBST for 5 min, the membrane was incubated at room temperature for 1 h with a secondary antibody. The bands were visualized using enhanced chemiluminescence.

### Plasmid and siRNA transfection

Plasmids were transfected with Lipofectamine 2000 transfection reagent (Invitrogen, California, USA), and the operation was followed by the manufacturer’s instructions. For NOLC1 or TRF2 knockdown, cells were transfected with small interfering RNA (siRNA) duplexes using RNAiMax transfection reagent (Invitrogen, California, USA) following the manufacturer’s instructions. The following siRNA sequences were used: TRF2: #1: 5′-GCUGCUGUCAUUAUUUGUA-3′ and #2: 5′-CCAGAAGGAUCUGGUUCUUTT-3′; NOLC1: #1: 5′-CACCAAGAAUUCUUCAAAU-3′ and #2: 5′-GCGAAAGUUACAGGCAAAU-3′. Control: 5′-UUCUCCGAACGUGUCACGU-3′.

### **C**hromatin immunoprecipitation

Cells were cross-linked with 1% formaldehyde for 10 min at room temperature. In all, 125 mM of glycine was added to quench the reaction. The cross-linked cells were then lysed in lysis buffer (1% SDS, 10 mM EDTA, pH 8.0, 50 mM Tris, pH 8.0, and protease inhibitor complex) (Roche, Basel, Switzerland) for 10 min at 4°C, sonicated for 45 s, and then centrifuged for 10 min at 14,000 × *g*. The supernatant was diluted with 4 (or 10) volumes of dilution buffer (1.2 mM EDTA, pH 8.0, 1.1% Triton X-100, 16.7 mM Tris, pH 8.0, and 300 mM NaCl) and incubated at 4 °C overnight with the corresponding antibody (2 mg of anti-TRF2 or anti-UBF), which was the corresponding amount of normal rabbit or mouse IgG and 20 ml of Protein-G Sepharose 4 Fast Flow beads. The beads were washed once with wash buffer 1 (0.1% SDS, 1% Triton X-100, 2 mM EDTA, pH 8.0, 20 mM Tris, pH 8.0, and 300 mM NaCl), wash buffer 2 (0.1% SDS, 1% Triton X-100, 2 mM EDTA, pH 8.0, 20 mM Tris, pH 8.0, and 500 mM NaCl), wash buffer 3 (500 mM LiCl, 1% NP-40, 1% Na-deoxycholate, 1 mM EDTA, and 10 mM Tris, pH 8.0), and washed twice with wash buffer 4 (1 mM EDTA, 10 mM Tris, pH 8.0). DNA fragments were elution and reversal at 65 °C overnight in 1% SDS, 0.1 M NaHCO_3_, 0.5 mM EDTA, pH 8.0, 20 mM Tris, pH 8.0, 10 mg of DNase-free RNase (Roche, Basel, Switzerland), or 0.5 M NaOH. For DNA extraction, a QIAquick PCR Purification kit (Qiagen, Dusseldorf, Germany) was used. The primers used were supplied in Table S3.

### Cell-cycle analysis

For cell-cycle analysis, cells were cultured to 60–80% confluency, then were treated with ActD or serum starvation for 12–24 h, and were washed twice in PBS, trypsinized, and were fixed in 75% ice-cold ethanol overnight, and incubated with 20 µg/ml DNase-free RNase A at 37 °C for 30 min. The cells were stained with propidium iodide (PI; 50 µg/ml; Sigma, CA, USA) for 15 min, and flow cytometry was conducted. Cell-cycle profiles were analyzed using Multi Cycle AV software.

### Statistical analysis

The data were statistically analyzed using the *t-*test. *P* < 0.05 was considered to represent a statistically significant difference. Data are representative of three independent experiments performed in triplicate.

### Other materials and methods

For a description of other materials and methods in this study, see Supplementary Information.

## Electronic supplementary material

Supplementary materials and figures
