# Supplementary material for: Nucleolar TRF2 attenuated nucleolus stress-induced HCC cell-cycle arrest by altering rRNA synthesis
Source: Cell Death Dis. 2018 May 3;9(5):518. doi: 10.1038/s41419-018-0572-3 (PMC5938709; doi:10.1038/s41419-018-0572-3)
Supplement: Supplementary file 1 — Supplementary materials and figures [file 41419_2018_572_MOESM1_ESM.pdf]

NOLC1 regulates the nucleolus accumulation of TRF2 and promotes rRNA transcription

Fuwen Yuan[1], Chenzhong Xu[1], Guodong Li[1], and Tanjun Tong[1],*

[1] Research Center on Aging, Department of Biochemistry and Molecular Biology, School of Basic Medical Sciences, Peking University, Beijing, China

* To whom correspondence should be addressed. Tel: +86 8280 2931; Fax: +86 8280 2931; E-mail: ttj@bjmu.edu.cn

Supplementary Information

Table S1: The primers used for TRF2 plasmids construction

Flag-TRF2-F	5'-CGGGATCCGCCACCATGGATTACAAGGATGACGACGATAA GGCCGCGGGAGCCGGGACG-3'
Flag-TRF2-R	5'-CCGCTCGAGTCATCAGTTCATGCCAAGTCTTTTCATGG-3'
Flag-△GAR-F	5'-CGCGGATCCGCCACCATGGACTACAAGGACGACGACGAC AAGGCGGGGGAGGCACGGCTGGAAGAG-3'
Flag-△GAR-R	5'-CCGCTCGAGTCATCAGTTCATGCCAAGTCTTTTCATGG-3'
Flag-△Myb-F	5'-CGGGATCCGCCACCATGGATTACAAGGATGACGACGATA AGGCCGCGGGAGCCGGGACG-3'
Flag-△Myb-R	5'-CCGCTCGAGTCATATATTGGTTGTACTGTCTTC-3'
Flag-△GAR&Myb-F	5'-CGCGGATCCGCCACCATGGACTACAAGGACGACGACGAC AAGGCGGGGGAGGCACGGCTGGAAGAG-3'
Flag-△GAR&Myb-R	5'-CCGCTCGAGTCATATATTGGTTGTACTGTCTTC-3'
Flag-△GAR&TRFH-F	5'CGCGGATCCGCCACCATGCTACAAGGACGACGACGACAAGGC CCATCCTGTTATCCAGAACT-3'
Flag-△GAR&TRFH-R	5'-CCGCTCGAGTCATCAGTTCATGCCAAGTCTTTTCATGG-3'

Table S2: The primers used for Real time PCR

NOLC1 F	5'-TTCCTGCGCGATAACCAACTC-3'
NOLC1 R	5'-CCTGTAACTTTCGCTCTGGGA-3'
ACTB-F	5'-ACCGCGAGAAGATGACCCA-3'
ACTB-R	5'-GGATAGCACAGCCTGGATAGCAA-3'
TRF2-F2	5'-ATCTGGTTCTTCCTACTCAAG-3'
TRF2-R2	5'-TACTCTGGCTGTCCTCCT-3'
46S-F	5'-GTCCGTCCTTCCGTTCGTCTT-3'
46S-R	5'-AGGAGGCGGGAACCGAAGAA-3'
32S-F	5'-GTCAGCGGAGGAGAAGAA-3'
32S-R	5'-CTCGATCAGAAGGACTTGG-3'
36S-F	5'-GCGGAGGTTTAAAGACCC-3'
36S-R	5'-CCAGACGAGACAGCAAAC-3'

Table S3: The primers used for CHIP.

P1-F	5'-GGAAGAGCCTACGCATTC-3'
P1-R	5'-GACAGAGTCAGAAGACAACC-3'
P3-F	5'-ACGGCTAGAGTGCAATGG-3'
P3-R	5'-GCCTGAGCAACATGGAGA-3'
P5-F	5'-TGCTTTCGTGCTTTCTTG-3'
P5-R	5'-CAAGCAAGCAAGCAAGAA-3'
P6-F	5'-TTCTCGACTCACGGTTTC-3'
P6-R	5'-AGAGCACGATCTCAAAGC-3'
P7-F	5'-TGTCAGGCGTTCTCGTCT-3'
P7-R	5'-ACCACATCGATCACGAAGAG-3'
P8-F	5'-AATTCGTTTCCGAGTCCC-3'
P8-R	5'-ACTCGCCAGAAAGGATCG-3'
P9-F	5'-GGTATATCTTTCGCTCCGAGTC-3'
P9-R	5'-AACCTCTCCAGCGACAGGT-3'

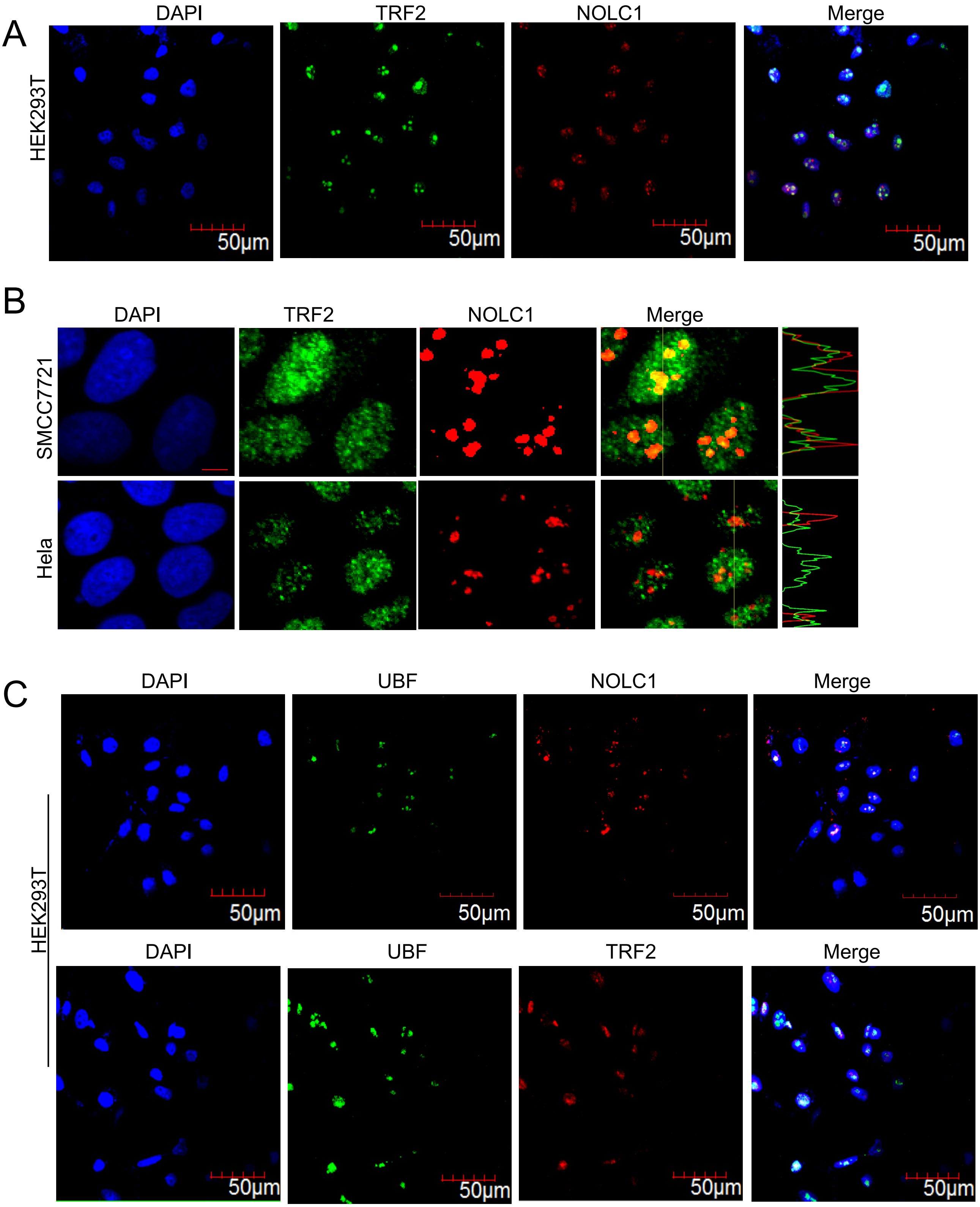

Figure S1 TRF2 colocalized with NOLC1 in the nucleolus. (A) Immunofluorescence analysis revealed that the nucleolar co-localization of TRF2 (green) and NOLC1 (red) in HEK293T cells in low magnification. (B) Immunofluorescence analysis revealed that the nucleolar co-localization of TRF2 (green) and NOLC1 (red) in SMCC7721, but not in Hela cells, scar bar, 5 μm. (C). The nucleolar co-localization of UBF (green) and NOLC1 (red) and UBF (green) with TRF2 (red) in HEK293T cells. Nuclei were visualized by DAPI (blue) staining.

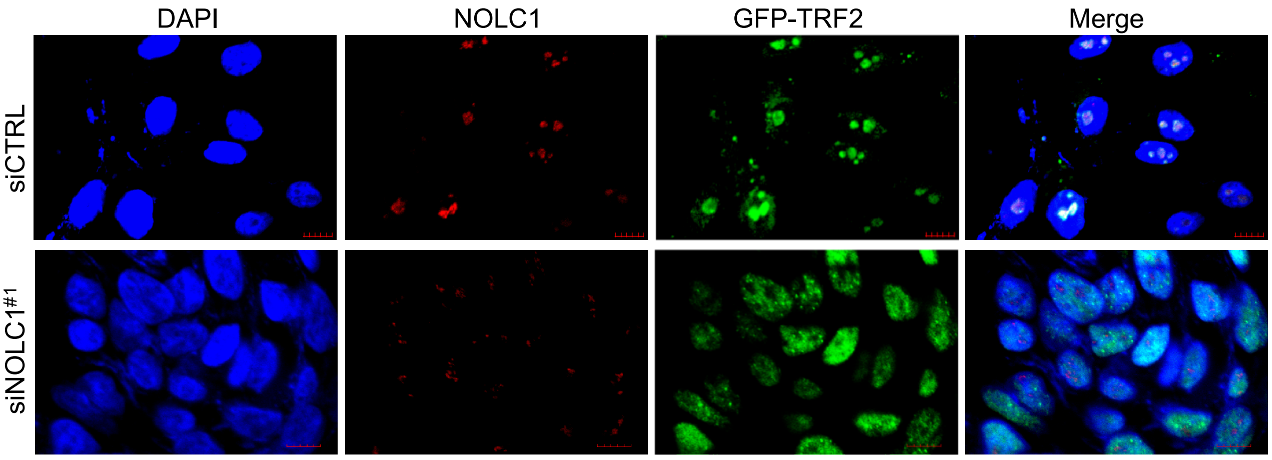

Figure S2 NOLC1 regulated the accumulation of TRF2 (green) in nucleolus. Re-destribution of TRF2 after NOLC1 (red) knockdown was analyzed with in HEK293T. Nuclei were visualized by DAPI (blue) staining. Scar bar 10 μm.

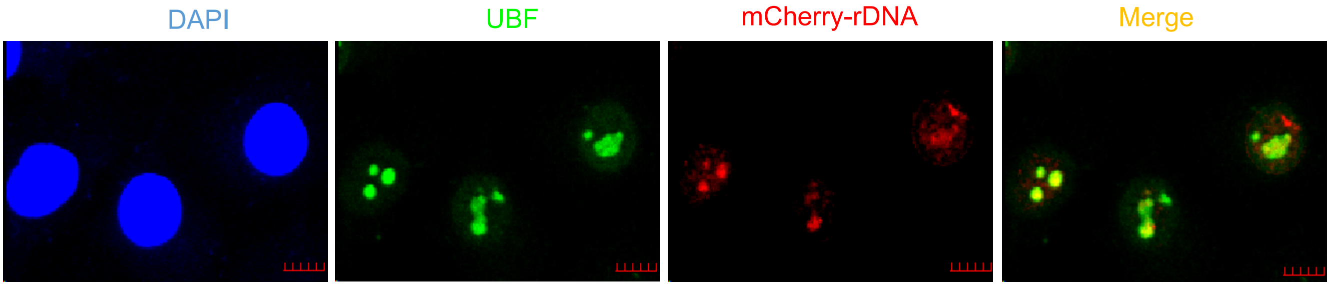

Figure S3 nucleolus marker UBF colocalized with mCherry-rDNA. Immunofluorescence analysis revealed that the nucleolar co-localization of UBF (green) and mCherry-rDNA (red) in HEK293T cells. Nuclei were visualized by DAPI (blue) staining. Scar bar 10 μm.

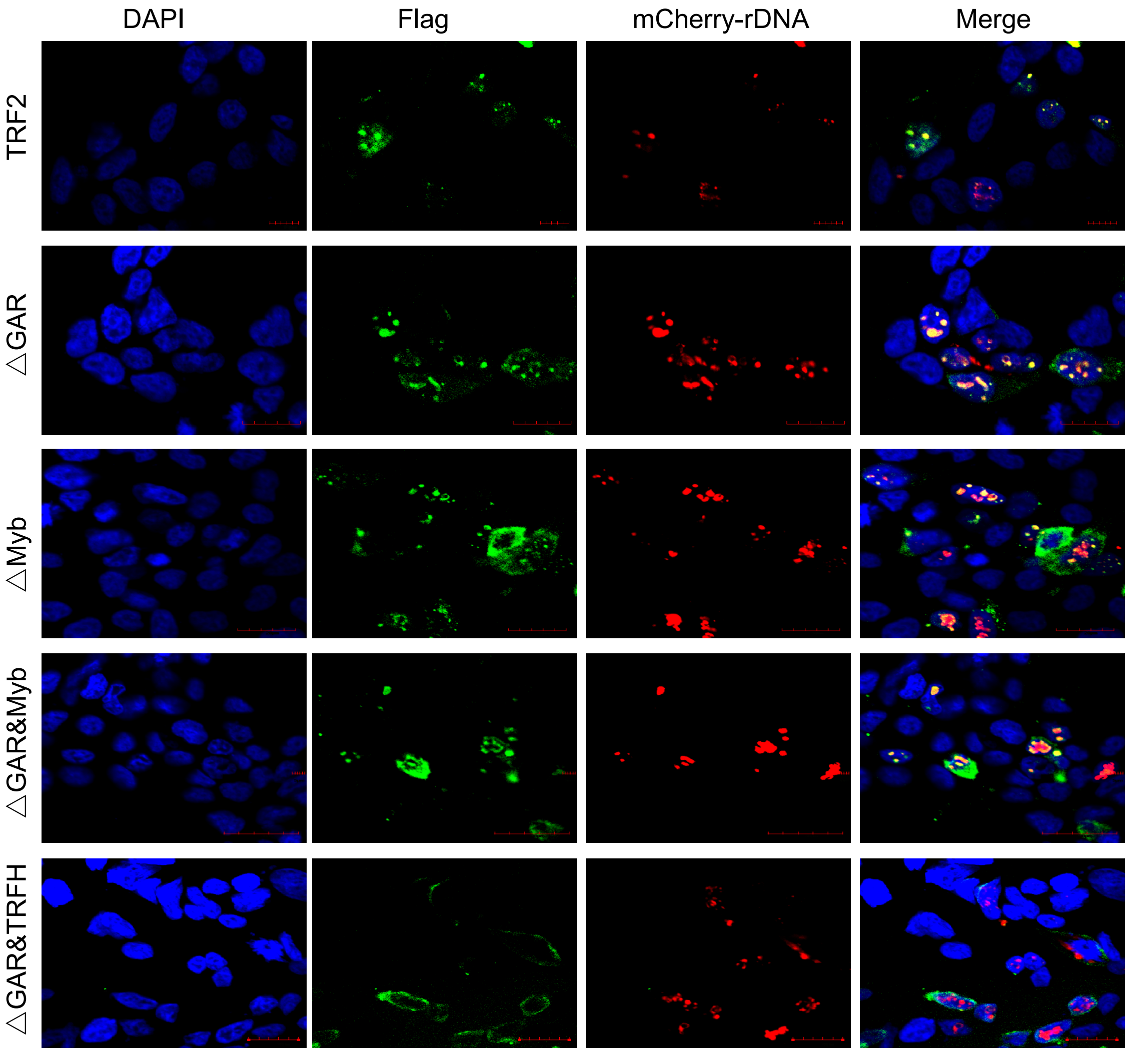

Figure S4 The domain deleted TRF2 disturbs rDNA advanced structure. Immunofluorescence visualized the protein expression of domain deleted TRF2 and the distribution the these TRF2 with rDNA. 293T cells were transfected with mCcherry-rDNA TALE system and both with Flag-TRF2 or the other Flag tagged mutant TRF2 for 48 h. The TRF2 (red) and rDNA foci (red) in nuclei of 293T were visualized with the indicated antibodies. Nuclei were visualized by DAPI (blue) staining. Scar bar 50 μm.

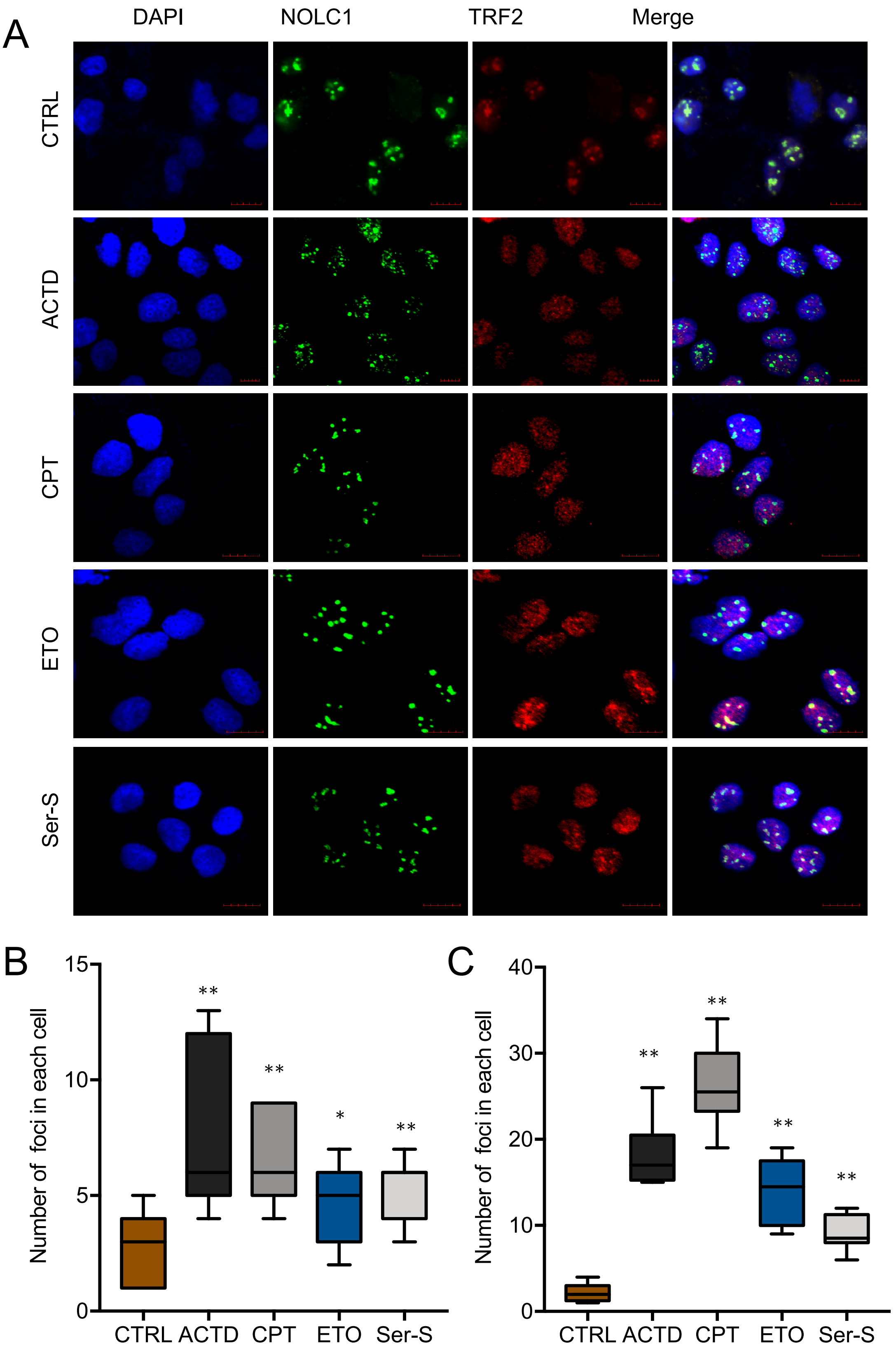

Figure S5 Nucleolar stresses dysregulate the co-localization of TRF2 and NOLC1 . (A) Immunofluorescence visualized the localization of TRF2 and NOLC1 in HEK293T cells after treated with (ActD, 5 nM, 6 h), Camptothecin (CPT, 20nM, 6 h), Etoposide (ETO, 40 nM, 6 h) and serum starvation (Ser-S, 0.1% FBS, 24 h). . Nuclei were visualized by DAPI (blue) staining. Scar bar 50 μm. (B) Statistics the foci number of NOLC1 in each cell visualized with immunofluorescence after different treatment. . (C)) Statistics the foci number of TRF2 in each cell visualized with immunofluorescence after different treatment.

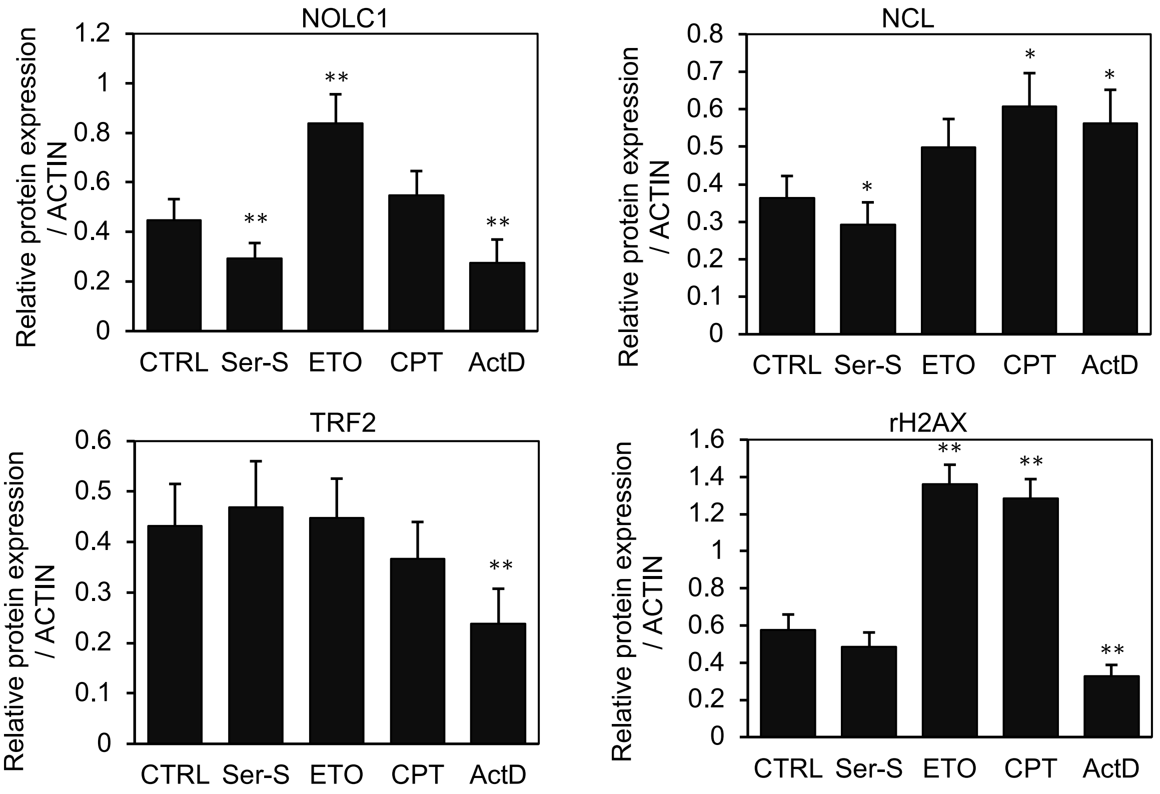

Figure S6 Statistics of the relative expression levels of NOLC1, NCL TRF2 and rH2AX proteins. Data are presented as the mean ± SD of three independently performed experiments. *; $p < 0.05$, **, $p < 0.01$.

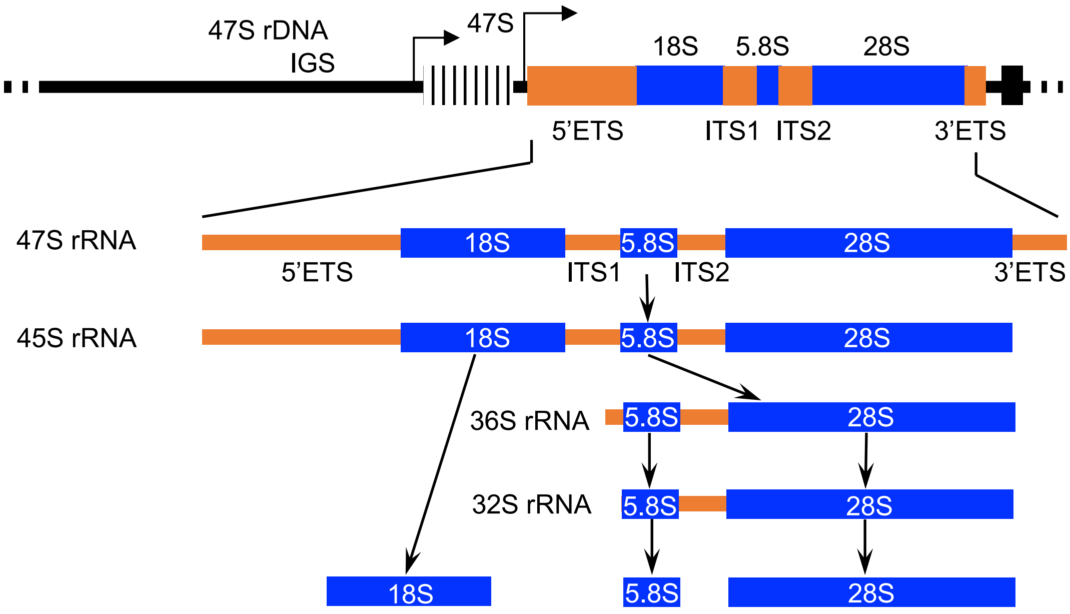

Figure S7 Schematic of rRNA processing.

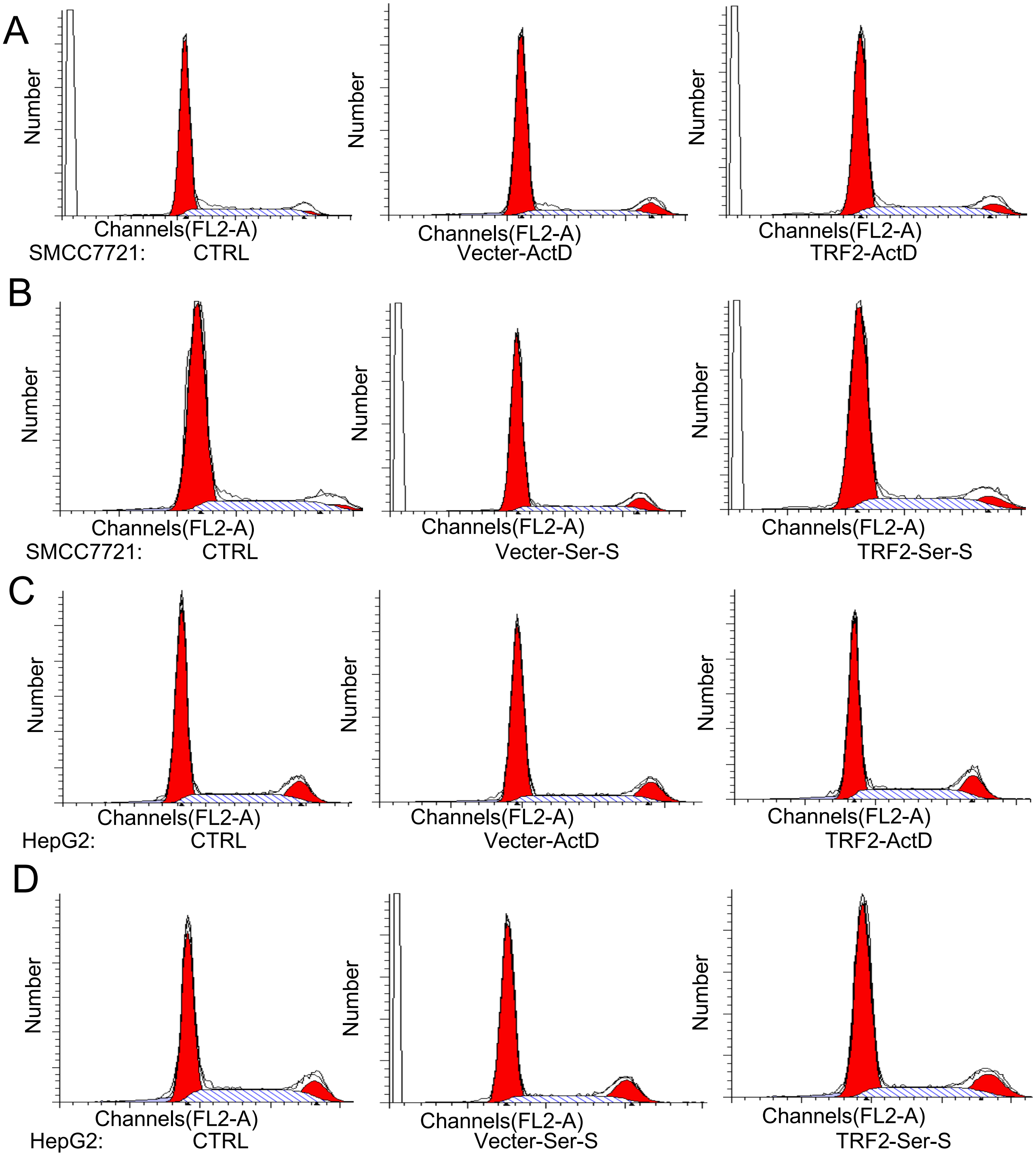

Figure S8 Overexpression of TRF2 rescued ActD and Serum starvation induced cell cycle arrest. (A) SMCC7721 cells were transfected with TRF2 for 48 h, and then the cells were treated with ActD for 10 h and flow Cytometry cell cycle analysis was performed. (B) SMCC7721 cells were transfected with TRF2 for 48 h, and were treated with DMEM (0.1%FBS) for 24h and flow Cytometry cell cycle analysis was performed. (C and D) HepG2 cells were used for cell cycle analysis and were performed as A and B. `
